# Severe Abdominal Pain Mimicking Appendicitis Caused by Imperforate Hymen: Case Report and Narrative Review

**DOI:** 10.3390/pediatric18010010

**Published:** 2026-01-13

**Authors:** Julia Kleina, Marcin Wieczorek, Karolina Markowska, Katarzyna Nierzwicka, Julia Leszkowicz, Agnieszka Szlagatys-Sidorkiewicz

**Affiliations:** 1 Faculty of Medicine, Medical University of Gdansk, M. Skłodowskiej-Curie 3a, 80-210 Gdańsk, Poland; 2 Nicolaus Copernicus Hospital in Gdańsk, Nowe Ogrody 1/6, 80-803 Gdańsk, Poland; 3 Children’s Surgery and Urology Ward, Nicolaus Copernicus Hospital in Gdańsk, Nowe Ogrody 1/6, 80-803 Gdańsk, Poland; 4 Department of Paediatrics, Gastroenterology, Allergology & Paediatric Nutrition Medical University of Gdańsk, Nowe Ogrody 1/6, 80-803 Gdańsk, Poland

**Keywords:** case report, abdominal pain, hymen, hematocolpos, hematometra

## Abstract

An imperforate hymen is a rare congenital genital anomaly causing menstrual blood retention during puberty. Treatment consists of a simple surgical incision of the hymenal membrane. We present a case of a 14-year-old girl who was admitted to the Emergency Department with severe lower abdominal pain mimicking appendicitis. Medical history revealed a lack of menses and several months of cyclic abdominal pain. Imaging diagnostics confirmed an imperforate hymen with hematometrocolpos. Hymenotomy was performed with full recovery without complications. An imperforate hymen should be considered in the differential diagnosis of abdominal pain in adolescent girls, especially without expected menstruation. Early recognition allows for prompt treatment and prevents complications.

## 1. Introduction

Abdominal pain is one of the most frequent symptoms in pediatrics. Typically, differential diagnosis encompasses the most common causes. Our case highlights the importance of expanding investigations to include rather rare causes. An imperforate hymen is a rare congenital genital anomaly, which in this case caused a common symptom—abdominal pain mimicking appendicitis.

## 2. Case Presentation

We report a case of a 14-year-old girl who presented to the Pediatric Emergency Department due to acute abdominal pain. Initially, the pain was located in the mesogastric region and then migrated to the right lower abdominal quadrant. The pain was aggravated by coughing. Before admission, she was vomiting repeatedly, had a subfebrile temperature, and specifically reported feeling constipated. The patient micturated properly without any dysuric symptoms. The child was in moderately good general condition. Upon palpation, the abdomen was painful with perceptible and palpable pathological resistance in the right lower quadrant and a positive Blumberg sign in the right lower quadrant. Apart from that, no abnormalities upon examination were found.

Due to suspicion of appendicitis, laboratory tests and abdominal ultrasound were carried out. The laboratory results showed leukocytosis at 12.7 × 10^9^/L (normal range: 4–10 × 10^9^/L) and neutrophilia at 9.96 × 10^9^/L (normal range: 1.8–7 × 10^9^/L), with a CRP value within the normal range (<5 mg/L) and without any other clinically significant deviations. The ultrasound revealed a dense, midline fluid collection in the retrovesical region of the lower pelvis, measuring approximately 123 mm × 78 mm × 85 mm, with a fluid–fluid level. The appearance was consistent with a distended vagina partially filled with hemolyzed blood. The uterus was adjacent to its upper pole, with intrauterine fluid forming a layer approximately 10 mm thick.

The overall appearance was suggestive of an imperforate hymen. A small amount of free fluid was also present in the right lower abdomen, between intestinal loops, with a layer thickness of up to 17 mm. Subsequently, the patient was consulted gynecologically. In her gynecological medical history, she reported that she never experienced any menstrual bleeding, but for a few months, she has experienced cyclical lower abdominal pain without bleeding, but prior medical evaluation was made. In addition, the patient’s mother and older sister menstruated at her age.

Upon examination, the patient’s physical development was as follows: height was between the 15th and 25th percentiles for age and sex, and BMI was at the 75th percentile for age and sex (according to WHO standards) [1,2]. The patient’s Tanner scale was B4/PH4. The inspection of external genitalia revealed an intact, bulging vaginal membrane with a blueish hue.

**Figure 1:**
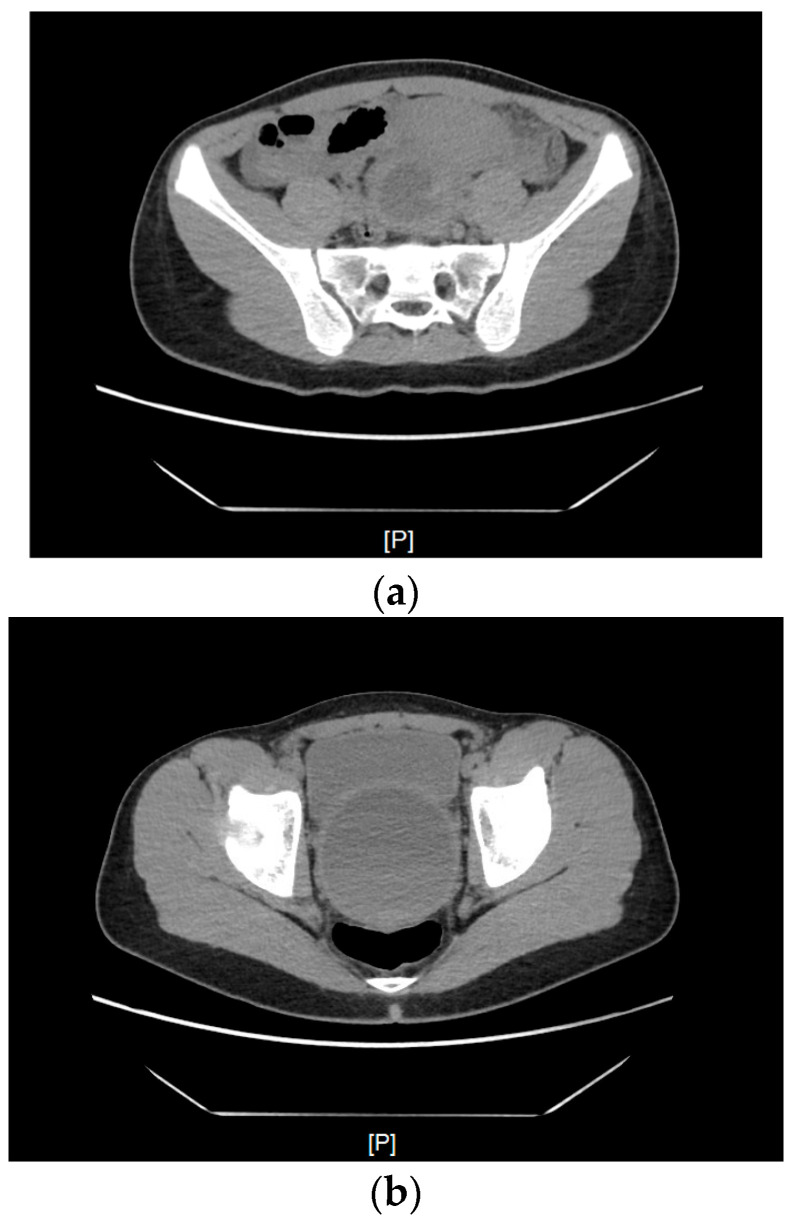

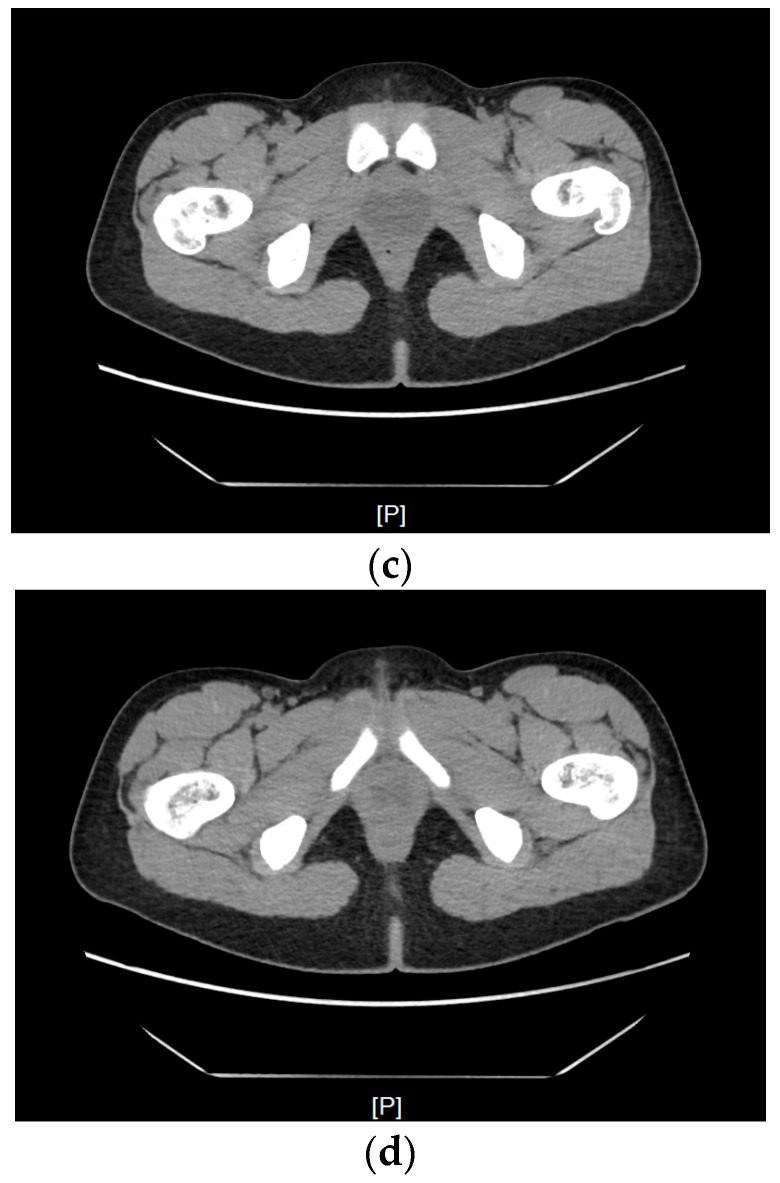
A CT scan of the uterus elevated superiorly, with fluid present in the uterine cavity, modeling the urinary bladder and rectum. (**a**) A scan at the level of the promontory. (**b**) From the superior part of the scan (anteroposterior): the urinary bladder, the uterus filled with hyperdense fluid, and posterior to it, the compressed rectum. (**c**) A scan at the level of the pubic symphysis. (**d**) A scan below the greater trochanter of the femur—a visibly enlarged vagina.

The patient was admitted to the Department of Pediatric Surgery and Urology. Before the hymenotomy, the patient underwent computed tomography (CT), which showed a significantly distended vagina filled with blood measuring approximately 78 mm × 81 mm × 130 mm. The uterus was elevated superiorly, with fluid present in the uterine cavity—features typical of hematometra. Enlarged parametrial venous plexuses were observed, along with edema of the adipose tissue along the parametrial ligaments (Figure 1).

Under general anesthesia, hymenotomy was carried out. A total of 500 mL of blood was evacuated without any complications. The patient was discharged the next day with a recommendation for gynecological care (due to risk of endometriosis). Following a one-month follow-up after the procedure, the patient reported general well-being and had her first menstrual bleeding. Occasionally, she complained of nonspecific abdominal pain. The patient is under the continuous care of a gynecologist.

**Figure 2:**
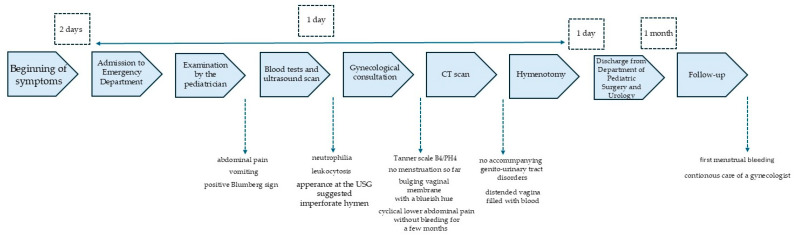
Timeline of diagnostic and therapeutic process.

The course of the diagnostic and therapeutic process is presented on a timeline (Figure 2).

## 3. Discussion

### 3.1. Literature Review Methodology

The review of the literature was performed using the PubMed and Google Scholar databases. We also used the AI tool OpenEvidence for additional background knowledge search. The general key terms searched within the registries were as follows: “imperforated hymen case report”, “hymenectomy”, “hymenotomy”, and “hydrocolpos”. The overall timeframe of the papers’ considered release was set from the year 2000 to the present. However, one older publication was included due to a greater number of patients involved. To be included, the specific publication had to address the problem of an imperforated hymen in general, other symptomatic genito-urinary abnormalities, or the diagnostic imagining of pelvic pathologies in the pediatric population.

### 3.2. Epidemiology of Imperforate Hymen

An imperforate hymen is an uncommon congenital genito-urinary abnormality. The incidence is estimated at 1 case per 1000–2000 female births or 0.05–0.1% [3,4,5,6]. The condition is usually diagnosed during adolescence (12–18 years) [3]. An imperforated hymen is usually diagnosed as an isolated condition [7], although some authors report the familial occurrence of occlusive and subocclusive hymenal variants. According to reported cases, both dominant and recessive models of inheritance are probable; thus, identifying an imperforated hymen should be followed by an evaluation of other female relatives [8,9]. Within the analyzed literature, no regionally increased incidence was noted. The most numerous cases occur in the 12–18-year-old age group (64.8%). This abnormality is much less frequently diagnosed at neonatal age (11%). In the literature, there are also a few cases of the detection of an imperforate hymen in those > 18 years old (4.7%) [5].

### 3.3. Symptomatology

A symptomatic presentation is characteristic of adolescent girls. Every month, menstrual blood accumulates behind the imperforate hymen and causes symptoms, of which the most frequently noted is abdominal pain. The vagina can undergo significant expansion—it may contain up to 2.5 L of blood [10]. Due to pressure on the surrounding organs in the pelvis, a dilated uterus may lead to disturbances in the urinary tract, such as dysuria, urinary tract infection, polyuria, or renal failure [11]. Neonates usually present with minimal clinical symptoms. The literature describes a case of neonates with a prenatally detected pelvic mass mimicking an ovarian cyst. In this case, hydrometrocolpos was diagnosed as the result of maternal estrogen stimulation [12,13]. Another neonatal patient presented with symptoms of intestinal obstruction resulting from mass effect [14].

An imperforate hymen is not the sole cause of hematocolpos, a condition characterized by a fluid-filled vaginal cavity. In differential diagnosis, congenital disorders like distal vaginal agenesis, transverse vaginal septum, and obstructed hemivagina and ipsilateral renal anomaly should be considered [15]. Apart from cases of imperforate hymen, instances of microperforate hymen have also been reported. This anomaly may remain undetected if menstrual blood can pass through the small opening. However, it can later result in dyspareunia or the inability to achieve vaginal intercourse in adulthood [16].

### 3.4. Etiology

During embryonic development, the upper two-thirds of the female genital tract (fallopian tubes, uterus, and upper part of vagina) form from the paramesonephric ducts. In contrast, the lower 1/3 part (lower part of the vagina) has an endodermal origin from the urogenital sinus [17]. In this junction, sinus-derived cells proliferate and form the sinovaginal bulb, the source for vaginal plate structure cells. The plate undergoes central epithelial degeneration, leading to the formation of the vaginal lumen. The remnant in this location is a thin mucosal membrane, the hymen. Physiologically, the hymen is perforated, and during puberty, menstrual blood from the uterus can freely flow through small fenestrations. If this stage of embryogenesis fails to proceed properly, a nonperforated membrane develops [15]. In view of the dynamic development of genetics and nuclear medicine over the past few years, the search for genes responsible for an imperforate hymen has been conducted. The probability of a genetic cause is supported by the familial occurrence of an imperforate hymen. J.R. Stelling et al. (2000) [9] reported a case of a 37-year-old woman who underwent prophylactic hymenectomy at 12 years of age (before symptom onset) because of family history: both her mother and her mother’s monozygotic twin (the patient’s maternal aunt) had required surgery for imperforate hymen during puberty [9]. Another report described a family in which monozygotic twin daughters and their mother each presented with imperforate hymen [18]. In both accounts, no genetic analyses were performed; therefore, a causative variant or locus could not be identified. A systematic review conducted by K.H. Lee et al. (2019) [5] proved that imperforate hymen is an isolated anomaly in 79.7% of cases. The rest of the cases were concomitant with other genital tract abnormalities, such as a transverse vaginal septum, vaginal atresia, double genital system [19], or anorectal malformation [20].

### 3.5. Diagnostic Imaging

MRI is instrumental in the evaluation of pelvic abnormalities, especially Müllerian duct disorders. Studies demonstrate that it reaches an overall accuracy of up to 97%, compared to 87% for a CT scan [21]. A recent scientific study reports on the growing role of 3D ultrasound, whose effectiveness is comparable to MRI [22]. This method has the potential for further development in the coming years.

In our institution, urgent MRI or 3D ultrasound is not available in an emergency setting, with typical waiting times of several days to at least one week, precluding its use in this case. Although MRI would have been preferable in an adolescent due to the absence of ionizing radiation, CT was performed after ultrasonography to swiftly confirm the diagnosis and exclude other accompanying genito-urinary disorders or other severe causes of abdominal pain and support operative planning within a clinically necessary timeframe. The decision to proceed with urgent surgery the following day was made by a multidisciplinary team (pediatric surgery—urology, gynecology, and pediatrics). Radiation exposure was considered, and the CT acquisition followed dose optimization principles (ALARA) [23]. During the CT procedure, the patient received 55 mL of iodine contrast agent (Omnipaque 350 mg/mL), to be precise, 19.25 g of iodine. According to ACR/SPR—American College of Radiology i Society for Pediatric Radiology, the recommended dosage is 1.5–2.0 mL/kg [24]. In conclusion, our patient (who weighed 51 kg at the time of CT scan) obtained the lowest possible dosage of iodine contrast, which was consistent with the recommendations of the pediatric scientific society. It is important to mention that, before the CT scan, we performed laboratory tests and marked the level of creatinine 0.6 (mg/dL) [normal range = 0.57–0.87 mg/dL]. This outcome enabled us to make decisions about contrast administration. After the supply of contrast, intravenous fluids were administered.

Additionally, the patient presented while traveling abroad, and symptom severity did not allow for safe discharge for a delayed outpatient MRI or referral to another center.

### 3.6. Treatment

Imperforate hymen is treated surgically. According to the literature, the procedure should be at best performed on asymptomatic patients during puberty, as vaginal tissue is then estrogenized, promoting proper healing [3,7]. There are several methods of imperforate hymen correction. The two most common treatments are hymenotomy and hymenectomy [5]. During hymenotomy, cross-shaped or U-shaped incisions are made, sparing the urethra, allowing for the outflow of retained liquid. Some authors also suggest performing an annular incision using an electrocautery ball-shaped probe, which preserves the hymenal membrane with no postoperative hymenal closure [3,4]. The second most common method—hymenectomy—can be described as hymenotomy extended by the excision of the hymenal membrane. After the initial incision (usually cross- or U-shaped), the excessive hymenal mucosa is then excised, and the remaining mucosal edges are approximated using absorbable sutures, securing proper hemostasis [7].

### 3.7. Outcomes

Complications related to an imperforate hymen can be classified as early or late. In the first group, there are complications secondary to the compression of the enlarged uterus, such as hydronephrosis and renal failure or obstruction of the gastrointestinal tract [25,26]. In the literature, postoperative pelvic infections are also described [27,28]. Late-onset complication manifests as restenosis of the hymenal membrane requiring reoperation [29]. One retrospective study indicated imperforate hymen and secondary hymenectomy as risk factors for significant obstetrical and gynecological outcomes, defined as more prevalent cesarean deliveries, more frequent infertility treatment, and dyspareunia [30]. Moreover, due to hematocolpos accompanying an imperforate hymen, there is a higher possibility that retrograde blood flow can cause endometriosis [31].

### 3.8. Limitations

Some limitations must be discussed. We do not have previous medical history/documentation about the diagnostic investigation concerning amenorrhea from the patient, because the patient did not undergo any medical evaluation. Due to a lack of possibility at the time of diagnosis in the Emergency Department, we did not perform an MRI; instead a CT scan was performed.

## 4. Conclusions

This case underlines several practical issues that are highly relevant for clinical practice, especially in the emergency setting. Our case shows that in adolescent girls presenting with acute lower abdominal pain, clinicians should routinely take a structured gynecological and endocrine history. This includes explicit questions about breast and pubic hair development, onset and pattern of menses, cyclic pelvic pain, and any suggestion of bleeding beyond absent visible menstruation. 

This report also illustrates the need to balance everyday diagnostic workflows with radiation protection. In many centers, CT performed for suspected appendicitis is the most available modality in the Emergency Department. However, whenever possible, ultrasound and MRI should be preferred as first-line imaging to minimize radiation exposure in adolescents.

In comparison with the existing literature, this case adds a pragmatic perspective on how such anomalies may be detected and managed when they present atypically and relatively late, in the context of a common surgical suspicion rather than a planned gynecologic work-up. Previous reports usually focus on the anatomical variant and ideal imaging algorithms.

This case also shows the practical role of a multidisciplinary team (pediatric surgery, pediatric gynecology, pediatrics) in proceeding to timely surgery. Taken together, these aspects highlight the value of systematic attention to menstrual history and the pragmatic use of imaging in the Emergency Department and may help clinicians reduce the risk of delayed diagnosis in similar presentations.

## Data Availability

The data presented in this study are available on request from the corresponding author.

## References

[1] WHO (2007). BMI-for-Age (5–19 Years).

[2] WHO (2007). Height-for-Age (5–19 Years).

[3] Abdelrahman H.M., Jenkins S.M., Feloney M.P. (2025). Imperforate Hymen. StatPearls.

[4] Klasa-Mazurkiewicz D., Wydra D., Milczek T., Emerich J. (2005). Urinary Retention Secondary to an Imperforate Hymen in a 16-Year-Old Patient—A Case Report and a Review of the Literature. Ginekol. Prakt..

[5] Lee K., Hong J., Jung H., Jeong H., Moon S., Park W., Jeong Y., Song S., Suk Y., Son M. (2019). Imperforate Hymen: A Comprehensive Systematic Review. J. Clin. Med..

[6] Jarmoliński T., Marciniak H., Grochocki J. (2018). Hematocolpos as a rare cause of acute urinary retention in a 14-year-old girl. Standardy Medyczne/Pediatria.

[7] Berger-Chen W., Anne-Marie E., Oelschlager A., American College of Obstetricians and Gynecologists’ Committee on Adolescent Health Care (2019). Diagnosis and Management of Hymenal Variants. Obstet. Gynecol..

[8] Watrowski R., Jäger C., Gerber M., Klein C. (2013). Hymenal Anomalies in Twins—Review of the Literature and Case Report. Eur. J. Pediatr..

[9] St elling J.R., Gray M.R., Davis A.J., Cowan J.M., Reindollar R.H. (2000). Dominant Transmission of Imperforate Hymen. Fertil. Steril..

[10] Sofoudis C., Sarantis N.-D., Salvanos G. (2025). Imperforate Hymen Mimicking Hematocolpos: A Case Report and Mini Review of the Literature. Obstet. Gynecol. Int. J..

[11] Lahfaoui M., Benhaddou H., Ettaybi F. (2020). Hematocolpos on Imperforated Hymen and Acute Urinary Retention: A Rare Disease About Seven Observations and Literature Reviews. J. Gynecol. Res. Obstet..

[12] Tegene D., Assefa T., Edris A. (2023). Neonatal Hydrometrocolpos Secondary to Imperforate Hymen Presented with Acute Urinary Retention: Case Report. Res. Rep. Neonatol..

[13] Podleśna M., Zgodzińska M., Sieńko C., Brodowska J., Torres A., Rybojad B., Lejman M., Osemlak P. (2025). Imperforate Hymen with Renal Complications? A Case of a 2-Day-Old Patient with Hydrometrocolpos. J. Pre-Clinical Clin. Res..

[14] Jouza M., Rejdova I., Cintula L., Jouzova A., Jabandziev P. (2024). Hydrocolpos Causing Bowel Obstruction in a Preterm Newborn: A Case Report. Matern. Health Neonatol. Perinatol..

[15] Tanitame K., Tanitame N., Urayama S., Ohtsu K. (2021). Congenital Anomalies Causing Hemato/Hydrocolpos: Imaging Findings, Treatments, and Outcomes. Jpn. J. Radiol..

[16] Ferrarini O.M.F., Munhoz L.O., Simões R.S., Cezarino P.Y.A., Mieli M.P.Â., Margarido P.F.R., Guida F.J., Baracate E.C. (2014). Microperforated Hymen: A Case of Delayed Diagnosis. Autops. Case Rep..

[17] Arbuckle J.L., Hoover K.H. (2016). Development of the Female Reproductive Tract and Associated Anomalies. Curr. Treat. Options Pediatr..

[18] Chua B.H.E., Amin Z., Ng Y.P.M. (2024). Familial Occurrence of Imperforate Hymen in Premature Monozygotic Twins and Their Mother: A Case Report and Literature Review. Front. Pediatr..

[19] Shaw L.M.A., Jones W.A., Brereton R.J. (1983). Imperforate Hymen and Vaginal Atresia and Their Associated Anomalies. J. R. Soc. Med..

[20] Khedkar K., Lamture Y.R., Lohia S., Tayade H.A. (2023). A Female Infant with Rectovestibular Fistula and Imperforate Hymen. J. Indian Assoc. Pediatr. Surg..

[21] Harringa J.B., Bracken R.L., Markhardt B.K., Ziemlewicz T.J., Lubner M., Chiu A., Birstler J., Pickhardt P.J., Reeder S.B., Repplinger M.D. (2021). Magnetic resonance imaging versus computed tomography and ultrasound for the diagnosis of female pelvic pathology. Emerg. Radiol..

[22] Qin C., Lee P., Luo L. (2025). Comparison between 3D-Enhanced Conventional Pelvic Ultrasound and Magnetic Resonance Imaging in the Evaluation of Obstructive Müllerian Anomalies and Its Concordance with Surgical Diagnosis. J. Pediatr. Adolesc. Gynecol..

[23] European Commission (2018). Radiation Protection No 185: European Guidelines on Diagnostic Reference Levels for Paediatric Imaging.

[24] American College of Radiology, American Society of Emergency Radiology, Society of Computed Body Tomography and Magnetic Resonance, Society of Pediatric Radiology (2019). ACR-ASER-SCBT-MR-SPR Practice Parameter for the Performance of Pediatric Computed Tomography (CT).

[25] Gherai R., Chitulea P. (2020). Hematohydro-Hysterocolpos with Vaginal Atresia.

[26] Tedyanto C.P., Dewi S., Santoso F.I., Ere M.A.P., Oeylex K.R. (2024). A Rare Case Report of a Congenital Imperforate Hymen Causing Obstructive Uropathy and Constipation in an 11-Year-Old Girl. Int. Med Case Rep. J..

[27] Wong J.W.H., Siarezi S. (2019). The Dangers of Hymenotomy for Imperforate Hymen: A Case of Iatrogenic Pelvic Inflammatory Disease with Pyosalpinx. J. Pediatr. Adolesc. Gynecol..

[28] Grimstad F., Strickland J., Dowlut-McElroy T. (2019). Management and Prevention of Postoperative Complications in a Neonate with a Symptomatic Imperforate Hymen. J. Pediatr. Adolesc. Gynecol..

[29] Dhande R.P., Singh R.K., Patwa P.A., Mishra G.V. (2021). A Case of Recurrent Hematocolpos Posthymenectomy in a Pubertal Girl with Transverse Vaginal Septum. J. Datta Meghe Inst. Med. Sci. Univ..

[30] Amitai E., Lior Y., Sheiner E., Saphier O., Leron E., Silberstein T. (2018). The Impact of Hymenectomy on Future Gynecological and Obstetrical Outcomes. J. Matern.-Fetal Neonatal Med..

[31] Lamceva J., Uljanovs R., Strumfa I. (2023). The Main Theories on the Pathogenesis of Endometriosis. Int. J. Mol. Sci..

